# Design and Performance Evaluation of Biomimetic Suction Cups Inspired by the Abalone Muscular Foot

**DOI:** 10.3390/biomimetics11020118

**Published:** 2026-02-05

**Authors:** Lingmi Wu, Yi Fang, Guoniu Zhu

**Affiliations:** 1 Academy of New Energy Equipment, Zhejiang College of Security Technology, Wenzhou 325016, China; 19086214@zjcst.edu.cn; 2 School of Automation and Intelligent Sensing, Shanghai Jiao Tong University, Shanghai 200240, China; 3 Academy for Engineering and Technology, Fudan University, Shanghai 200433, China

**Keywords:** biomimetic suction cup, abalone muscular foot, groove–seal structure, finite element analysis, interfacial stress

## Abstract

This study addresses the limited adsorption efficiency of traditional vacuum suction cups used in applications such as unmanned aerial vehicles (UAVs) and robotic systems. Inspired by the adhesion mechanism of the abalone muscular foot, we propose a novel suction cup design. The design incorporates optimization of the groove geometry, groove distribution, and the positioning of the sealing ring. To assess the mechanical performance of these designs, finite element analysis (FEA) is employed. A prototype exhibiting the most promising simulation results is fabricated and subjected to tensile testing. The experimental results show strong agreement with the simulation outcomes, thereby validating the accuracy and reliability of the FEA. The biomimetic suction cup demonstrates superior adsorption performance compared to the baseline design. Specifically, the maximum von Mises stress is reduced by 5.9%, the maximum pressure is increased by 498%, and the maximum frictional stress rises by 498%. Moreover, the maximum sliding distance is reduced by 38%, while the maximum total circumferential deformation is increased by 21%. This innovative design enhances the stress distribution across the bottom surface of the suction cup, mitigates inward edge contraction, and delays the communication between the inner cavity and the external environment, thereby improving the overall adsorption efficiency of the suction cup.

## 1. Introduction

Vacuum suction cups are widely used because of their high efficiency, low cost, and environmental friendliness. They have been extensively applied in robotic systems operating on vertical surfaces, such as unmanned aerial vehicles (UAVs) and wall-climbing robots [1,2]. Recently, a biomimetic suction cup inspired by biological adhesion mechanisms has attracted increasing attention as a promising alternative to conventional vacuum suction cups, suffering from limited sealing reliability and reduced adsorption efficiency under complex conditions [3]. It also exhibits considerable potential for practical applications. Inspired by biological attachment systems, particularly the abalone muscular foot, biomimetic suction cups with non-smooth surface morphologies have been proposed. Previous studies indicate that such bio-inspired structural features can improve contact conformity and mechanical behavior at the interface, thereby enhancing adsorption and sealing performance [4]. Consequently, abalone foot–inspired suction cups have become an active research topic, while further investigation into their structural optimization and underlying mechanisms is still required.

Extensive research has been conducted on the adhesion mechanisms of aquatic and soft-bodied organisms such as lumpsucker, octopuses, clingfish, and abalones. Existing studies demonstrate that biological adhesion relies not solely on suction, but integrates compliance, friction, and microstructures to achieve robust attachment. Notably, organisms maintain adhesion during motion and disturbance, underscoring the effectiveness of their sealing interfaces. These mechanisms offer key insights for designing biomimetic suction cups with enhanced sealing and performance in wet or complex environments [5,6,7,8].

The strong adhesion capabilities of biological tissues have inspired extensive research on biomimetic suction cup design for robotic applications. Studies have investigated passive suction for climbing robots [9], octopus/squid-inspired structures for manipulation [10,11,12], and soft-rigid hybrid designs for aerial/climbing applications [13,14]. Structural optimizations like grooves and multilayer designs further enhance sealing and stability [15]. While these works highlight the importance of geometry, compliance, and surface features, most focus on singular structures or specific scenarios. The combined influence of surface morphology and sealing design on stress, sliding, and sealing reliability remains underexplored. This study integrates abalone-inspired grooves and a muscular foot-like sealing ring to systematically improve adsorption performance.

In underwater robotics, the requirements for the sealing and adhesion performance of biomimetic suction cups have become increasingly demanding. Follador et al. [16] proposed a dielectric elastomer-driven biomimetic suction cup. The soft underwater actuator produced an adhesion pressure of up to 6 kPa within 300 ms. Xi et al. [17] designed a biomimetic octopus suction cup with surface grooves. Its adhesion force increased by 12.2% in air and 25.2% underwater compared with a standard suction cup. Sandoval et al. [18] reported that the papillae structures of the clingfish sucker improve sealing through fluid adhesion and wet friction, providing insights for suction devices. Tsujioka et al. [19] created biomimetic structures with strong underwater adhesion. Their results showed that combining soft and hard materials is key to strong adhesion. Li et al. [20] designed an adaptive suction cup, attaching to 3D surfaces in both dry and wet conditions. Its adhesion strength changed dynamically by more than 30 times. Wang et al. [21] fabricated a biomimetic octopus suction cup using 3D-printed Zr^4+^-coordinated hydrogels. The suction cup achieved adhesive strengths of 48.46 kPa in air and 57.19 kPa underwater, overcoming the limits of traditional materials in reversible high-adhesion applications.

As the application scope of suction cups expands, particularly in complex environments such as rough and irregular surfaces, single-structure designs—while effective under simpler conditions—can no longer meet the growing demands for higher adhesion force and adaptability. Bio-inspired suction cups, with their innovative multi-structure designs, are better equipped to address these challenges, delivering superior performance in demanding practical applications. Ditsche et al. [22] developed the first suction cups inspired by the northern clingfish. These cups achieved an adhesion strength up to 70 kPa on rough surfaces. Tsukagoshi et al. [23] proposed a novel soft hybrid-structure suction cup, suitable for porous surfaces and irregular objects. The prototype, with an outer diameter of 55 mm and a weight of 18.8 g, was able to generate an adhesive force of 80 N in the vertical direction and 60 N in the shear direction on porous walls. Wang et al. [24] developed a stiffness-gradient soft suction cup inspired by the octopus sucker. It increased adhesion force by 45.2% on curved surfaces. Cong et al. [25] designed a groove-type suction cup based on *Sinogastromyzon szechuanensis*, enhancing the adsorption force by up to 71.22%.

In bio-inspired suction cup research, computational modeling and simulations are widely employed. Mei et al. [26] validated structural improvements through static finite element analysis in ANSYS. Xi et al. [17] used FEM to show that non-smooth deformation enhances adhesion by increasing contact area. Dixit et al. [27] combined FEA and FDM printing to demonstrate stress dispersion via internal cavities. Xu et al. [28] simulated how bionic grooves affect contact pressure and friction.

Although extensive research has been carried out on the morphology and adhesion mechanisms of suckers in organisms such as octopuses, leeches, and abalones [5,6,7,8], these studies have mainly focused on understanding biological adhesion and developing biomimetic models with good performance. Current research on abalones has primarily examined the adhesion ability of the muscular foot on smooth surfaces, the factors contributing to adhesion force, and the microstructural features of the foot surface [6,17]. However, few studies have applied the surface structural characteristics of the abalone muscular foot to improve existing suction cups. In particular, the effects of changes in morphology and the number of structures on the bottom surface of the suction cup on its adhesion performance have not been fully studied. Research on the biomimetic design of sealing and adhesion structures inspired by the abalone muscular foot remains limited. Most related research focuses on biological or medical fields, while in UAV applications, despite extensive studies on multi-rotor aerodynamics, the design of auxiliary suction cups for aerial tasks has received little attention.

Based on biomimetic principles, this study translates the surface morphology and sealing behavior of the abalone muscular foot into a parametric groove–seal architecture for suction cup design. Instead of merely mimicking the biological form, the groove width, depth, height, and angular distribution, as well as the sealing-ring geometry, are explicitly defined as tunable engineering parameters. A series of biomimetic suction cups are constructed and systematically evaluated using finite element analysis to quantify how these parameters regulate stress distribution, interfacial sliding, and cavity sealing. Comparative simulations and experiments are then conducted to identify the structure–function relationships governing adsorption performance.

The biomimetic suction cup structure with superior adsorption performance identified from the finite element analysis is selected for prototyping. A 3D-printed mold is used to fabricate the suction cup prototypes through casting. Tensile tests on the physical prototypes reveal the high-efficiency adhesion mechanism of the biomimetic suction cups and help determine the optimal structural parameters. The results show that, compared with the baseline smooth suction cup, the biomimetic suction cup reduces the maximum von Mises stress by 5.9%, increases the maximum pressure by 498%, and increases the maximum frictional stress by 498%. Moreover, the maximum sliding distance is reduced by 38%, while the maximum total circumferential deformation is increased by 21%. These results demonstrate that, relative to the baseline, the proposed abalone-inspired groove–seal design significantly improves the overall adsorption performance by enhancing stress distribution, sealing stability, and resistance to interfacial sliding, thereby supporting its potential for UAV and wall-climbing robotic applications.

## 2. Geometric Structure of the Biomimetic Suction Cups

In this study, a commercial vacuum suction cup with a top hook attachment is selected as the baseline geometric model. For numerical modeling, the hook and mounting structures are removed to focus the analysis on the adsorption interface. The suction cup has a nominal outer diameter of ϕ 55 mm. The dimension represents a common medium size in robotic vacuum gripping applications rather than a globally unified standard. A three-dimensional model of this geometry is established as the smooth baseline configuration for subsequent comparative analysis.

### 2.1. Baseline Alignment and Cross-Biomimetic Comparability

To achieve a meaningful comparison between the proposed biomimetic suction cup and existing research, the baseline geometry in this study is aligned with the model proposed by Xu et al. [29] in terms of geometry dimensions, material properties, and loading conditions. Based on a comparable suction cup configuration, this model has been widely used as a reference for performance comparison in studies on grooved and sealing-ring suction cup designs. This alignment strategy ensures that performance differences among models stem solely from the biomimetic surface structures, thereby enabling a fair and reproducible comparative analysis.

Building upon this baseline geometry, a series of biomimetic suction cup configurations were developed by incorporating abalone muscular foot-inspired non-smooth surface morphologies. All biomimetic designs maintained the same outer diameter, material parameters, and loading conditions as the baseline suction cup, thereby ensuring that any observed performance variations were solely attributable to the introduced surface structural modifications.

The abalone muscular foot exhibits distinct macroscopic folds and grooves along its anterior–posterior axis, which undergo cyclic contraction and expansion during attachment and locomotion [30,31]. When subjected to external disturbances, such as an upward pulling force, the tentacles retract into the body, and the surface of the foot forms irregular striated grooves and folds. This surface texturing actively suppresses detachment and enhances the adhesion performance of the abalone. Simultaneously, the peripheral papillae contract inward, pressing tightly against each other to form an annular sealing structure around the outer edge of the foot. This mechanism allows the abalone foot to function like a suction cup, enabling the organism to remain firmly attached to rocky substrates during movement or feeding, thereby resisting wave impact and maintaining stability. When the ambient conditions return to normal, the tentacles re-extend and the papillae return to their extended state [32,33,34,35,36,37]. This deformation enables the foot to achieve large-area conformal contact on complex substrates, establishing a stable macroscopic sealing interface that supports adhesion [5,38]. Detaching an abalone from its substrate first requires breaking the annular sealing structure formed by the contracted papillae of its muscular foot. As a result, the grooves suppress premature detachment and enhance overall adhesion performance. The groove structures promote controlled deformation of the contact surface under external loading, which helps maintain conformal contact and delays seal failure. At the same time, groove-induced contraction facilitates the formation of an annular sealing region at the periphery, improving cavity sealing stability and reducing interfacial sliding.

Although the foot also possesses hierarchical micro-/nanoscale fibrous structures and may contribute to wet adhesion through van der Waals forces and capillarity [39,40], these microscopic mechanisms operate at scales and through material systems that are difficult to translate directly into the negative-pressure sealing and manufacturing frameworks of elastomeric vacuum suction cups. Therefore, this study adopts a functional biomimetic abstraction approach, focusing on translating the macro-scale folding/grooving behavior and the peripheral annular sealing features of the abalone foot into an engineered groove–sealing suction cup structure. Based on these biological observations, two key biomimetic features were extracted: (1) the grooved surface morphology formed under stretching, and (2) the annular sealing structure formed by peripheral papillae of the foot. These features were realized in the suction cup design as a groove network and a peripheral sealing ring, respectively.

### 2.2. Suction Cup Surface Morphology Design

**Figure 1:**
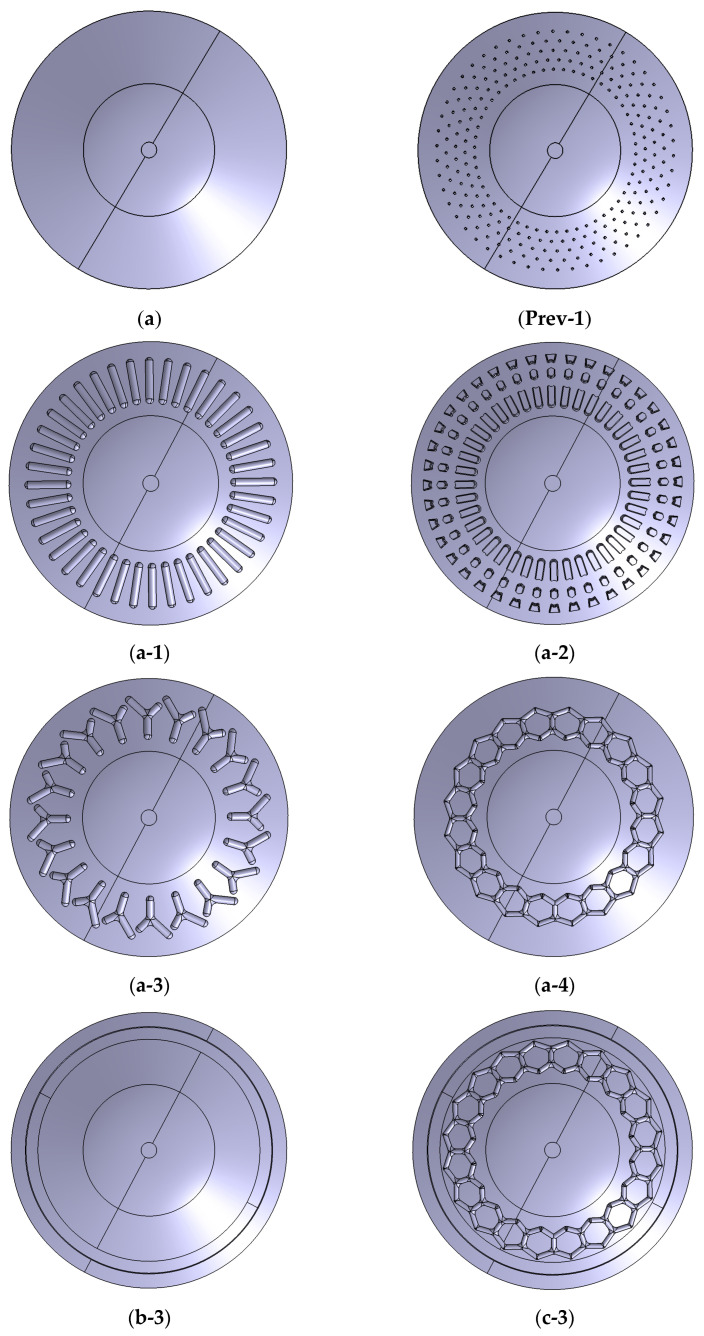
Surface Morphologies of the Baseline, reference, and representative biomimetic suction cups. (**a**) Smooth Baseline Suction Cup; (**Prev-1**) Xu-type reference suction cup with circular hole array (×50); (**a-1**) Biomimetic Suction Cup with Slotted Groove Array; (**a-2**) Biomimetic Suction Cup with Radial Groove Array; (**a-3**) Biomimetic Suction Cup with Herringbone Groove Array; (**a-4**) Biomimetic Suction Cup with Hexagonal Groove Array; (**b-3**) Biomimetic Suction Cup with Sealing Ring (width 2.5 mm); (**c-3**) Biomimetic Suction Cup with Hexagonal Groove Array and Sealing Ring (width 2.5 mm).

**Figure 2:**
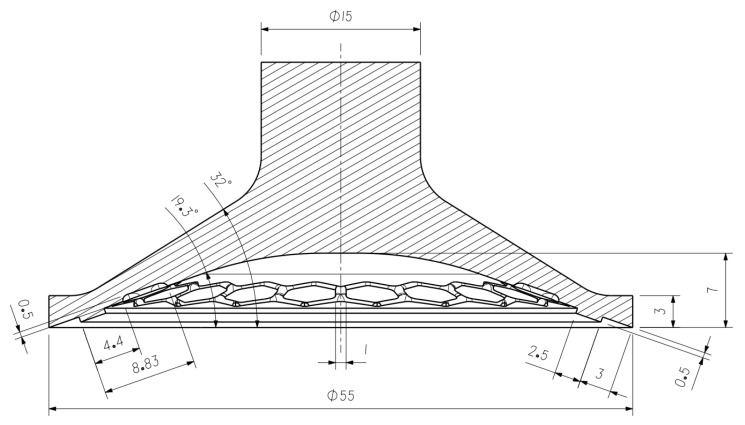
Dimensional Diagram of the Biomimetic Suction Cup (c-3).

The biomimetic abstraction was guided by considerations of scalability and manufacturability. Although the abalone foot possesses hierarchical structures—including micro-fibers (length: 35–45 μm, diameter: 0.5–4 μm) that may facilitate wet adhesion [41]—these microscopic features are not directly transferable to the design of dry, negative-pressure elastomeric suction cups. Consequently, our design intentionally focuses on the macro-scale functions: the groove morphology and the peripheral sealing ring. Based on these two features, ten biomimetic configurations (a-1–a-4, b-1–b-3, and c-1–c-3) are proposed, together with a smooth baseline suction cup (a) and Xu-type reference models (Prev-1/Prev-2) reproduced from Xu et al. [29] for benchmarking, as listed in Table 1 and Figure 1. The ten biomimetic configurations are further grouped into three categories for three-dimensional modeling and analysis, and the geometry of the configuration with the best overall performance is shown in Figure 2.

**Figure 3:**
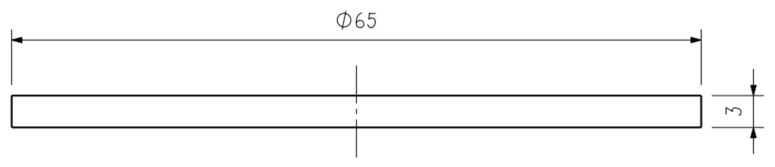
Dimensional Diagram of the Adsorption Plate.

**Figure 4:**
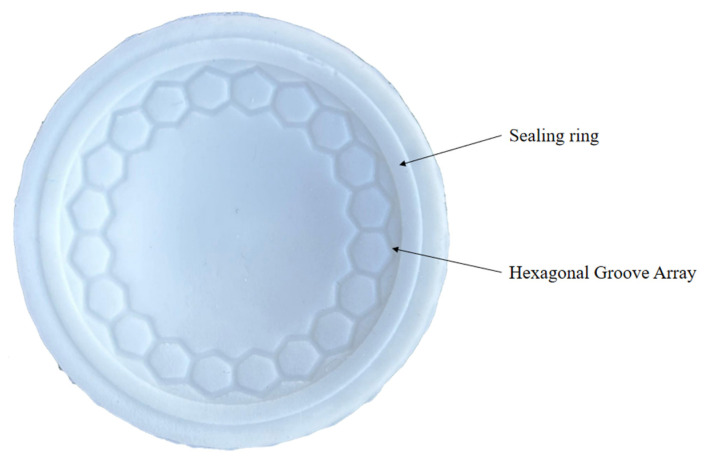
Prototype of the Biomimetic Suction Cup (c-3) with Hexagonal Groove Array and Sealing Ring.

As listed in Table 1, the first category includes suction cups with only groove structures on the working surface. The abalone muscular foot shows clear annular characteristics with stripe and cross-stripe patterns. According to these features, four types of groove structures are designed: slotted, radial, herringbone, and hexagonal, as illustrated in Figure 1(a-1–a-4). The groove dimensions are determined from the morphological parameters of the abalone muscular foot and proportionally scaled to match the baseline suction cup, labeled as a-1, a-2, a-3, and a-4.

The second category includes suction cups with only an annular sealing structure on the working surface, as shown in Figure 1(b-3). Based on the bottom surface dimensions of the baseline suction cup and the papillae width of the abalone muscular foot, sealing rings with widths of 0.5 mm, 1.5 mm, and 2.5 mm are designed near the edge of the cup. These models are named b-1, b-2, and b-3. The third category combines the previous two designs. On the basis of the a-4 suction cup, sealing rings with widths of 0.5 mm, 1.5 mm, and 2.5 mm are added, forming models c-1, c-2, and c-3, as shown in Figure 1(c-3).

The adsorption plate is designed as a circular plate with a diameter of 65 mm. As shown in Figure 3, its initial thickness is set to 3 mm. As shown in Table 2, Model 3 serves as the baseline thickness configuration for subsequent finite element analysis. To systematically evaluate the influence of glass thickness on the adsorption performance of the biomimetic suction cup, comparative models with thicknesses of 1 mm, 2 mm, 4 mm, and 5 mm are established within the same geometric framework for parametric simulation studies.

The mold for the biomimetic suction cup (c-3) is fabricated using 3D printing, and the suction cup is formed by silicone casting, as shown in Figure 4.

## 3. Structural Analysis of the Biomimetic Suction Cup Based on the Finite Element Method

### 3.1. Fundamental Principles of Finite Element Analysis

The adsorption interaction between the biomimetic suction cup inspired by the abalone muscular foot and the adsorption plate can be described using an equilibrium equation based on continuum mechanics theory.

(1) $$\nabla •\sigma +f=\rho \frac{\partial^{2}u}{\partial t^{2}}$$

where ∇•σ is the divergence of the stress tensor, representing the distribution of internal forces within the material; ∇ denotes the divergence operator, describing the internal stress state of the material; σ is the Cauchy stress tensor; f is the body force per unit volume, accounting for externally applied forces such as pressure and gravity; $\rho \frac{\partial^{2}u}{\partial t^{2}}$ represents the inertial force per unit volume; ρ is the material density; *u* is the displacement vector; and $\frac{\partial^{2}u}{\partial t^{2}}$ represents the acceleration.

The adsorption process between the biomimetic suction cup inspired by the abalone muscular foot and the adsorption plate can be regarded as a quasi-static process. Under the static assumption, the acceleration is zero, and the internal and external forces are balanced. Therefore, the equilibrium equation can be simplified as follows:

(2) ∇•σ=0

In this study, to simplify the analysis, we assume f = 0, i.e., the effect of external body forces is neglected, allowing the focus to remain on the internal force distribution and deformation behavior of the biomimetic suction cup. The geometric relationship between strain and displacement of the suction cup can be expressed by the following geometric equation:

(3) ε=∇u

where *ε* denotes the strain tensor, representing the degree of material deformation; ∇*u* represents the displacement gradient, describing the spatial variation in the displacement field.

Through these governing equations, the finite element analysis (FEA) is able to simulate the deformation and stress distribution of the biomimetic suction cup under load, thereby providing a theoretical foundation for subsequent evaluation of its adsorption performance.

### 3.2. Finite Element Analysis Setup

**Figure 5:**
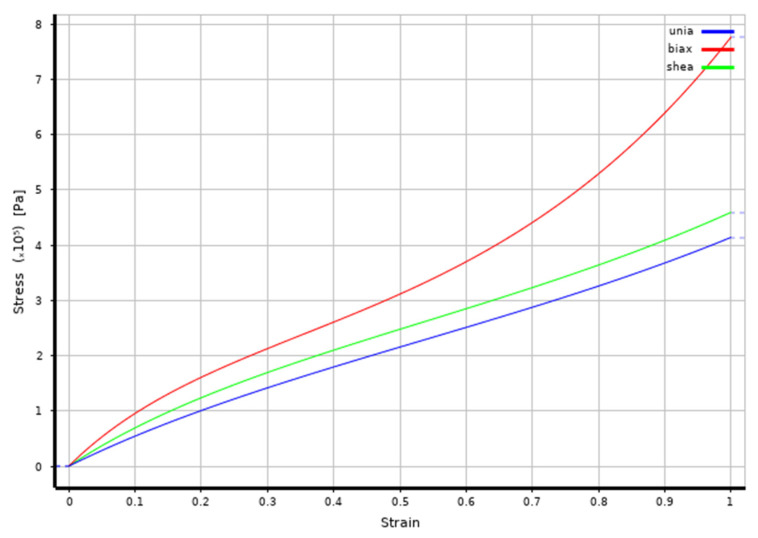
Third-Order Yeoh Hyperelastic Model Fitting Curve.

The baseline suction cup model is imported into the static structural analysis module of the FEA software (ANSYS 2020 R2) for mechanical simulation. The suction cup has a diameter of ϕ 55 mm, while the adsorption plate is configured with dimensions of ϕ 65 mm × 3 mm. The plate surface is larger than the suction cup to avoid boundary effects and to ensure accurate simulation of the adsorption behavior. The adsorption plate is made of glass with a density of 2.46 g/cm^3^, an elastic modulus of 68.9 GPa and a Poisson’s ratio of 0.23 [42,43,44,45]. The biomimetic suction cup material is silicone, with a density of 1.2 g/cm^3^, hardness of 25 A, tensile strength of 4.01 MPa, tear strength of 24 kN/m, and viscosity of 3.5 × 10^4^ Pa·s, providing significant deformation capability. To describe its nonlinear mechanical behavior, the third-order Yeoh hyperelastic model is applied [18,29,46,47]. The Yeoh model is a commonly used hyperelastic material model for describing the nonlinear behavior of rubber materials, particularly suitable for simulating the response of rubber materials under large deformations. The first, second, and third deviatoric material constants are set to C10 = 99.161 kPa, C20 = −1.604 kPa, and C30 = 1.065 kPa. These parameters define the stiffness and nonlinear response of the material under shear deformation. The silicone rubber is assumed to be fully incompressible, with the volumetric material constants D1 = 0 kPa^−1^, D2 = 0 kPa^−1^, and D3 = 0 kPa^−1^. Figure 5 shows the fitting curve of the third-order Yeoh hyperelastic model. During the adsorption process, the silicone suction cup primarily exhibits uniform deformation in the plane; therefore, a biaxial fitting curve is used to more accurately describe the material’s response.

For mesh discretization, the suction cup part uses 20-node quadratic solid elements (SOLID285) for free tetrahedral meshing, which is suitable for large deformation and hyperelastic material simulations. The glass plate is meshed with 8-node linear solid elements (SOLID185) for hexahedral meshing. The global mesh size is set between 0.8 and 1.2 mm to ensure computational efficiency. To improve the accuracy of the calculation in critical regions, a local mesh refinement strategy is implemented: First, the mesh is refined in key geometric regions, such as the grooves and sealing rings of the suction cup, with a size control of 0.3–0.5 mm, ensuring at least 3–4 layers of elements in the groove depth direction. Secondly, the mesh is refined at the contact interface, with the minimum element size smaller than 1/3 of the contact feature size to accurately capture the contact pressure distribution and deformation behavior.

Since the adsorption behavior of the suction cup involves contact interaction, the upper surface of the adsorption plate is defined as the target surface, and the conical surface at the bottom of the suction cup is defined as the contact surface. Frictional contact is defined between the suction cup and the adsorption plate, with a coefficient of friction of μ = 0.20. The upper surface of the adsorption plate is defined as the target surface, and the conical surface at the bottom of the suction cup is defined as the contact surface. The contact modeling employs the Augmented Lagrange method. The Normal Stiffness Factor is set to 1.0.

**Figure 6:**
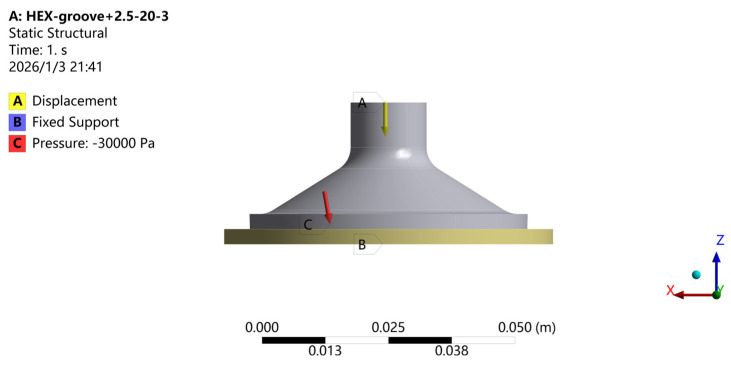
Schematic of the applied loading and boundary conditions. Note: The arrows indicate the applied displacement (A) and pressure loading (C), while the bottom surface (B) is defined as a fixed support. Symbols and number formats follow the default output of ANSYS Workbench.

To enable a comparative analysis between the biomimetic suction cups and the baseline suction cup, as well as to study the stress and strain distributions under adsorption conditions, the external load is simplified as a displacement constraint. The top surface of the suction cup is set as the displacement boundary with a displacement of −6 mm along the *Z*-axis, while the X and Y directions are kept free. A negative pressure of 30,000 Pa is applied to the inner surface of the suction cup [29,41]. A fixed constraint is applied to the bottom surface of the adsorption plate to fully restrict its motion. The applied loading and boundary conditions are shown in Figure 6.

### 3.3. Finite Element Analysis Results

**Figure 7:**
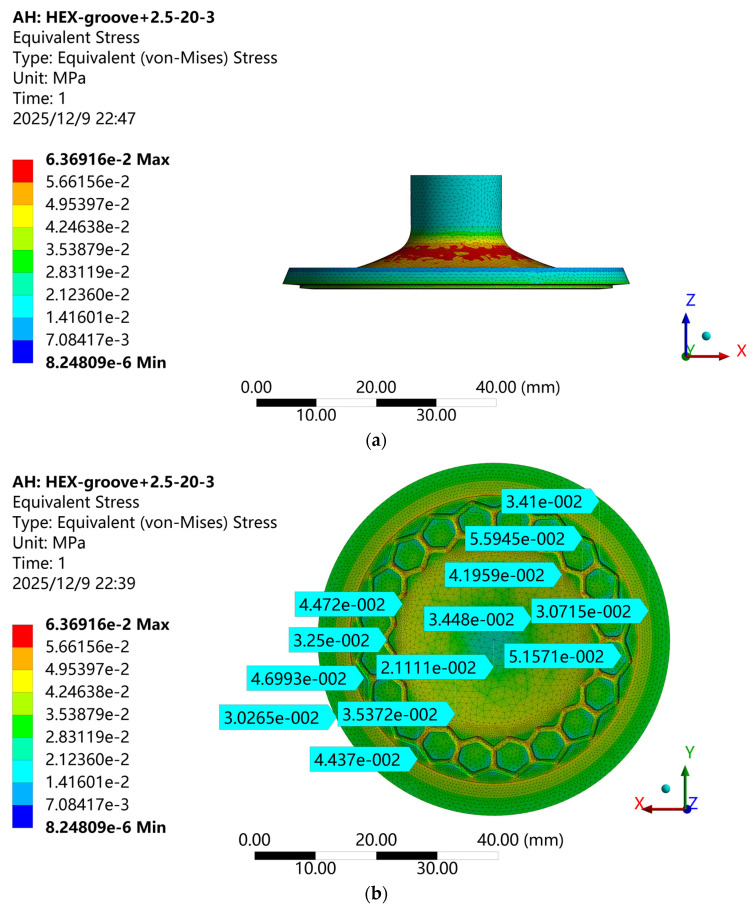
Von Mises Stress Contour of the Biomimetic Suction Cup (c-3). (**a**) Overall Von Mises Stress Distribution of the Biomimetic Suction Cup (c-3); (**b**) Von Mises Stress Distribution on the Working Surface of the Biomimetic Suction Cup (c-3). Note: The symbols and scientific notation in the figure follow the default output format of ANSYS Workbench.

After defining the loading and boundary conditions, finite element analyses are performed for both the baseline suction cup and the biomimetic suction cups with different structural morphologies. The simulations provide the equivalent (von Mises) stress distributions under the adsorption state, as shown in Figure 7. For the biomimetic suction cup (c-3), relatively high stress values appear on the working surface, but the maximum stress does not occur in this region. Instead, it is concentrated at the rounded corners on the outer non-working surface of the suction cup.

**Figure 8:**
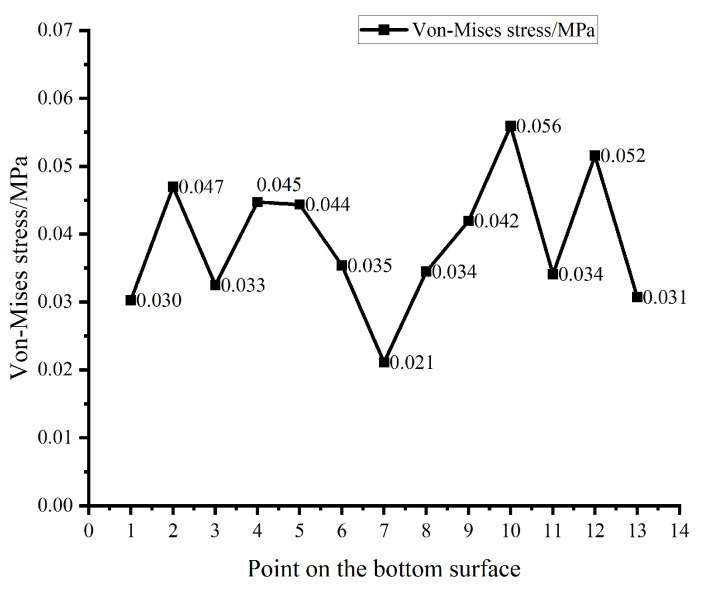
Von Mises Stress Curve of the Biomimetic Suction Cup (c-3).

To analyze the stress distribution at the bottom of the biomimetic suction cup (c-3), thirteen sampling points are selected along the diameter from one edge to the center and then to the opposite edge, based on the equivalent (von Mises) stress contour shown in Figure 7b. The von Mises stress values at these points are extracted, and the corresponding stress distribution curve is plotted, as shown in Figure 8. The results show that the stress is significantly higher in the hexagonal groove regions and lower in the smooth conical areas. The grooves increase the contact area between the suction cup and the adsorption surface, which results in higher localized stresses in the grooved regions. These structural features enhance the overall adhesion performance by improving the conformal sealing between the suction cup and the adsorption surface. Moreover, the stress distribution displays a clear radial gradient on the bottom surface of the suction cup. The edge region experiences higher stress, while the stress gradually decreases toward the center. A distinct annular stress concentration zone appears near the outer rim, located on the inner side of the sealing ring. Due to the high flexibility of the silicone rubber material, this localized peripheral stress concentration enhances tight adhesion between the suction cup and the adsorption plate, contributing to a stable and efficient sealing interface.

For suction cups, both adsorption efficiency and sealing performance strongly depend on the mechanical and geometric conditions of the contact interface. Therefore, the contact tool in the solution module is used to extract detailed data on interface behavior under the adsorption state. Specifically, the contact status, directional deformation, frictional stress, pressure, and sliding distance distributions are obtained and analyzed.

**Figure 9:**
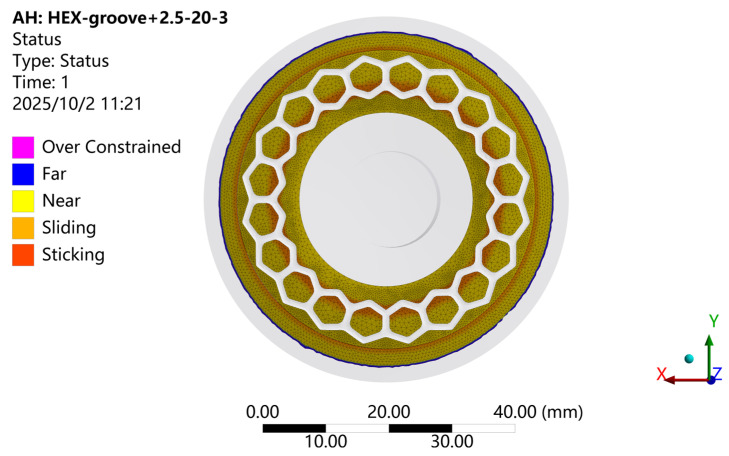
Contact interface status of the biomimetic suction cup (c-3) under the adsorption condition.

Figure 9 shows the contact interface status of the hexagonal-groove biomimetic suction cup (c-3) with a sealing ring under the adsorption condition. As illustrated, when the suction cup is compressed to its lowest position, the conical surface outside the sealing ring remains out of contact with the adsorption plate. This indicates that the sealing ring effectively maintains its sealing function. Noticeable sliding occurs along the inner edge of the sealing ring and within the inner regions of the hexagonal grooves, leading to outward expansion and deformation of the suction cup structure.

To investigate the variation law of the suction cup’s sealing area and analyze its adsorption performance, precise measurement of the deformation of the suction cup’s outer diameter after loading is required. During the compression of the suction cup, due to the uniform distribution of forces in the bottom region, the stress and strain at each point exhibit symmetrical distribution around the center of the suction cup. Therefore, the deformation displacement at each point along the same circumference at the edge of the suction cup is nearly identical. Based on this, when studying the deformation of the suction cup at its lowest position, the outer circumference of the suction cup is selected as the object for finite element analysis, and the deformation of this circumference is calculated using the Total Deformation analysis method in the ANSYS Workbench platform [48].

**Figure 10:**
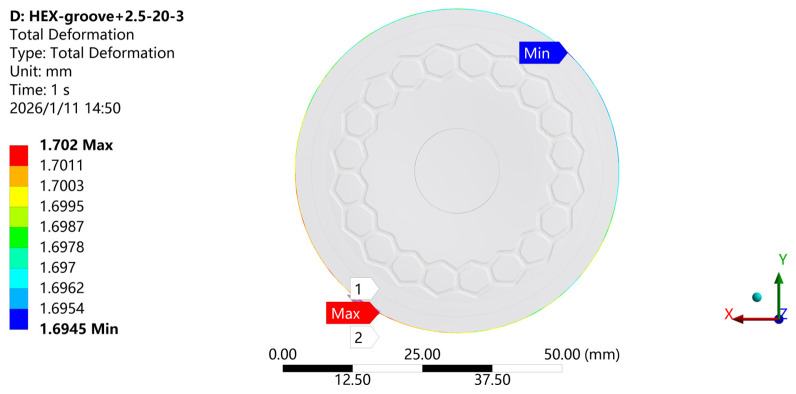
Total deformation contour of the biomimetic suction cup (c-3) at the outer circumference.

As shown in Figure 10, the results indicate that the deformation of the outer circumference of the suction cup is largely axisymmetric, suggesting that the deformation is relatively uniform along the circumference. Overall, the results demonstrate that the suction cup maintains a symmetric deformation behavior in most areas during its working state, indicating that the working state during the compression process is symmetric.

**Figure 11:**
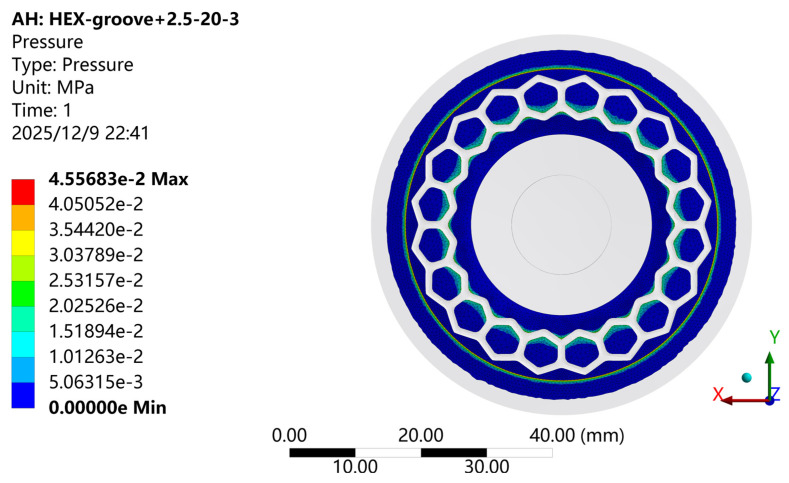
Pressure Contour of the Biomimetic Suction Cup (c-3).

**Figure 12:**
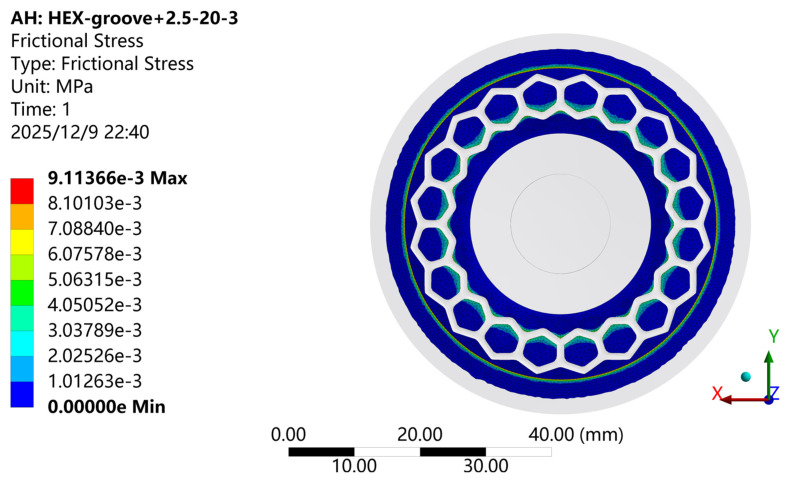
Frictional Stress Contour of the Biomimetic Suction Cup (c-3).

Figure 11 shows the pressure contour of the contact surface for the biomimetic suction cup (c-3) under the adsorption condition, and Figure 12 presents the corresponding frictional stress contour. As illustrated, the pressure and frictional stress distributions on the working surface of the suction cup show a high degree of consistency. Both exhibit an annular pattern radiating outward from the center of the suction cup. The overall trend increases first, then decreases, rises again to a secondary peak, and finally decreases toward the edge. The maximum values of both frictional stress and pressure are concentrated along the inner surface of the sealing ring. When the suction cup is fully compressed to its maximum deformation under the applied loading conditions, the conical region outside the sealing ring remains out of contact with the adsorption plate, confirming the critical role of the sealing ring in maintaining the sealed interface.

**Figure 13:**
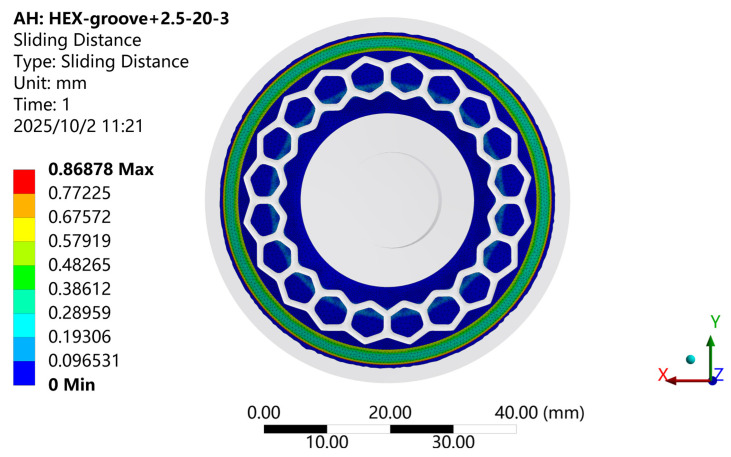
Sliding Distance Contour of the Biomimetic Suction Cup (c-3).

Figure 13 shows the contour of sliding distance variation for the biomimetic suction cup (c-3) under the adsorption condition. As illustrated, the sliding distance on the working surface exhibits an annular distribution pattern radiating outward from the center of the suction cup. The magnitude of sliding distance follows a complex oscillatory trend: it increases first, then decreases, rises again, and finally diminishes toward the periphery after reaching a second peak. The maximum sliding distance occurs along the outer edge of the sealing ring, indicating that the circumferential boundary of the sealing structure experiences the greatest relative motion during compression.

### 3.4. Comparison Between the Biomimetic Suction Cups and the Baseline Suction Cup

Finite element analyses are conducted for the baseline suction cup and the ten biomimetic suction cups using identical loading and boundary conditions.

**Figure 14:**
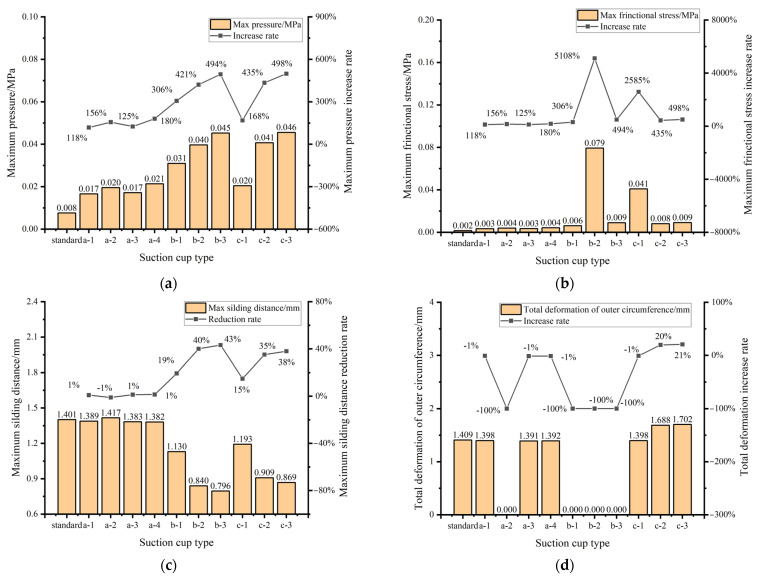
Comparison between the Biomimetic Suction Cups and the Baseline Suction Cup. (**a**) Comparison of Pressure between the Biomimetic Suction Cups and the Baseline Suction Cup; (**b**) Comparison of Frictional Stress between the Biomimetic Suction Cups and the Baseline Suction Cup; (**c**) Comparison of Sliding Distance between the Biomimetic Suction Cups and the Baseline Suction Cup; (**d**) Comparison of Total Deformation of Outer Circumference between the Biomimetic Suction Cups and the Baseline Suction Cup.

A comparative evaluation among the four shapes is performed with consideration of the morphological characteristics of each suction cup design to isolate the effect of groove topology on the response. As shown in Table 3 and Figure 14, compared to the smooth baseline suction cup, the four groove shapes increase the maximum contact pressure and maximum frictional stress by approximately 118% to 180%, while the change in maximum sliding distance is relatively small (approximately −1% to +1%). Among these, the hexagonal groove (a-4) achieves the highest pressure and frictional stress, while the radial groove (a-2) exhibits the largest sliding distance. As shown in Table 3 and Figure 14, the difference in maximum von Mises stress among the four groove shapes do not exceed approximately 5%, indicating that the performance differences are primarily determined by the groove topology rather than changes in the overall structural stress levels.

Notably, the configurations with an annular sealing structure (Group b) exhibit a maximum total circumferential deformation on the order of 10 × 10^−4^ mm, demonstrating the significant stiffening effect of the sealing ring, restricting overall deformation. The radial groove (a-2) also exhibited the same characteristic in the deformation of the outer circumference.

As illustrated in Figure 14a, the biomimetic suction cups show a substantial increase in maximum pressure. Among the designs, suction cup (c-3) demonstrates the most significant improvement, with an increase of 498% compared with the baseline suction cup. This improvement indicates a stronger seal between the suction cup and the surface, which can enhance the suction ability and adhesion performance. The increase in pressure reflects the potential for more effective adhesion, particularly when maintaining a stable connection under stress. Figure 14b shows that the maximum frictional stress also increases across all biomimetic designs. Suction cup (b-2) achieves the highest enhancement, with an increase rate of 5108%. As shown in Figure 14c, the maximum sliding distances of suction cups (a-1), (a-3), and (a-4) are similar to those of the baseline suction cup, while the maximum sliding distance of the (a-2) suction cup is greater than that of the baseline suction cup. In contrast, suction cup (b-3) exhibits the greatest reduction in sliding distance, with a decrease of 43%. Figure 14d shows that suction cup (c-3) displays the largest outer diameter deformation, with a 21% increase relative to the baseline suction cup.

**Figure 15:**
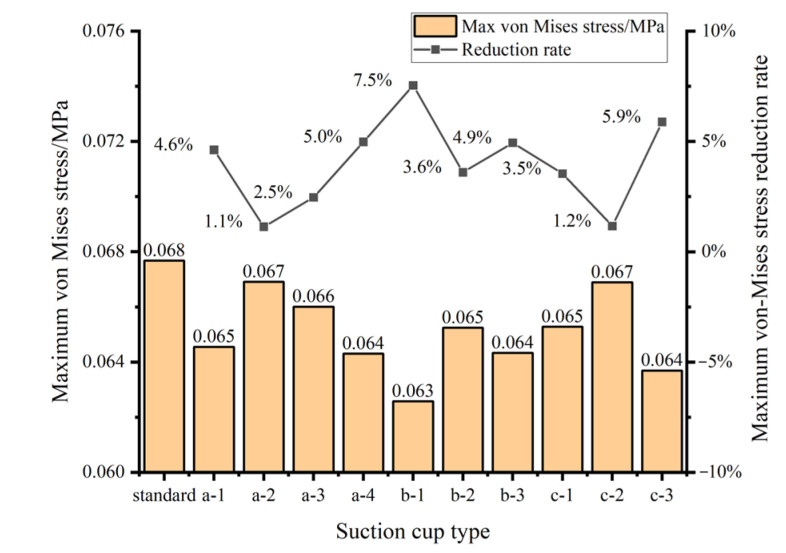
Comparison of maximum von Mises stress between the biomimetic suction cups and the baseline suction cup.

A comparative analysis of the maximum von Mises stress is conducted between the baseline suction cup and the biomimetic suction cups. As shown in Figure 15, all biomimetic suction cups exhibit a reduction in maximum von Mises stress compared with the baseline design. Among them, the biomimetic suction cup (b-1) shows the lowest value, with a 7.5% decrease relative to the baseline suction cup. In contrast, the suction cup (c-2) presents the highest von Mises stress among the biomimetic designs; however, its value is still 1.2% lower than that of the baseline model.

Based on these results, the biomimetic suction cup (c-3) demonstrates the best overall adsorption performance among the tested designs. This superior performance arises from the combined effect of the groove structure and the sealing ring, which promotes a more uniform stress distribution across the contact interface and effectively suppresses interfacial sliding, thereby enhancing sealing stability. Quantitatively, compared with the baseline suction cup, the c-3 design shows a 5.9% reduction in maximum von Mises stress, a 498% increase in maximum contact stress, a 498% increase in maximum frictional stress, a 38% reduction in maximum sliding distance, and a 21% increase in maximum outer circumferential deformation. Together, these improvements indicate an enhanced ability to maintain a stable and efficient seal under external loading, which is critical for achieving high adsorption performance.

### 3.5. Effect of Glass Thickness on Suction Cup Performance

To evaluate the effect of different glass thicknesses on the performance of the biomimetic suction cup, simulations were conducted for five different thicknesses ranging from 1 mm to 5 mm. The table below presents the key performance metrics of the suction cup for different glass thicknesses, including maximum von Mises stress, outer circumferential deformation, maximum frictional stress, maximum contact stress, and maximum sliding distance.

As shown in Table 4, with the increase in glass thickness, there are some variations in the suction cup’s outer circumferential deformation, frictional stress, and sliding distance. The variation in maximum von Mises stress across different thicknesses is small, remaining nearly constant, with only a slight difference between 1 mm and 5 mm, but the change is not significant. Pressure remains stable, with almost no noticeable differences, and all thickness designs perform similarly in this regard. The variation in frictional stress is also relatively steady across the different thickness designs, with the 5 mm thickness showing slightly higher frictional stress, but the difference is minimal. Compared to other thickness designs, the 4 mm thick glass design exhibits the smallest sliding distance (0.752 mm), indicating improved stability. The total circumferential deformation of the suction cup shows little variation across different thicknesses, with the smallest difference observed between the 3 mm (original thickness) and the other thicknesses.

Overall, although the differences in performance metrics across different glass thicknesses are relatively small, the 4 mm thickness performs best in terms of sliding distance, suggesting that it may be an ideal design choice. The 3 mm (original thickness) still maintains a relatively balanced performance, so in subsequent biomimetic suction cup designs, the 3 mm thickness will continue to serve as the baseline for further optimization.

## 4. Tensile Test Comparison of the Biomimetic Suction Cup

### 4.1. Tensile Test Conditions and Experimental Setup

In this study, the biomimetic suction cup (c-3) with the best simulation performance is selected for the tensile test. The mold model for the suction cup is designed in two parts: an upper mold and a lower mold. Both molds are fabricated using 3D printing. A nylon rope is pre-threaded through the top of the upper mold to facilitate the subsequent tensile test. The transparent silicone rubber and curing agent are mixed at a mass ratio of 20:1 and stirred for 3–5 min. The mixture is then injected into the mold cavity and left to cure for 4 h. After curing, the biomimetic suction cup prototype is obtained, as shown in Figure 4.

**Figure 16:**
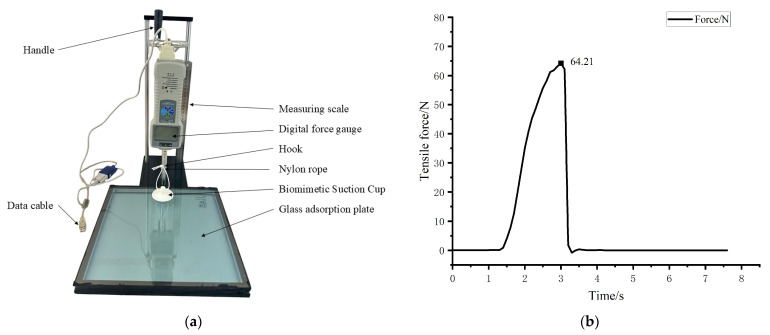
Tensile test Comparison of the Biomimetic Suction Cup. (**a**) Experimental Setup for the Tensile Test of the Biomimetic Suction Cup; (**b**) Tensile Force—Time Curve for the Biomimetic Suction Cup (c-3).

As shown in Figure 16a, a ZP-100N digital force gauge (accuracy: 0.01 N; brand: AILIGU, Shenzhen, China) is used to perform the tensile test on the biomimetic suction cup. To measure the adsorption force on a glass surface, the force gauge is first connected to a computer via a data cable and reset to zero. The nylon rope attached to the top of the suction cup is then fixed to the hook of the force gauge. The height of the force gauge is adjusted by rotating the handle until the nylon rope is in a natural, unstressed position. The suction cup is then compressed by 6 mm using a reference scale to ensure full contact between its bottom surface and the glass adsorption plate. This compression expels the air between the two surfaces, allowing the suction cup to adhere to the glass plate. Finally, the handle is rotated, and the suction cup is pulled upward through the hook until it is completely detached from the glass plate.

### 4.2. Tensile Test Results

During the tensile test, the tensile force applied to the suction cup is continuously recorded by the force gauge. As shown in Figure 16b, at the beginning of the experiment, the tensile force increases rapidly as the force gauge is gradually raised, leading to a sharp rise in the adhesion force of the suction cup. As the suction cup deforms under the upward tensile force, the vacuum suction from the sealed chamber and the edge compression together resist the applied force. The tensile force curve shows a short fluctuation followed by a further increase until it reaches its peak value. The suction cup detaches completely from the glass plate when the adhesion force is no longer sufficient to balance the upward tensile force. At this moment, the tensile force curve drops sharply below zero, with the suction cup suspended from the hook. The maximum value recorded during the test is taken as the adhesion force of the suction cup on the glass plate. Fifty repeated tensile tests are carried out on the biomimetic suction cup, and the average of the fifty measurements is used as the final result, as shown in Table 5.

**Figure 17:**
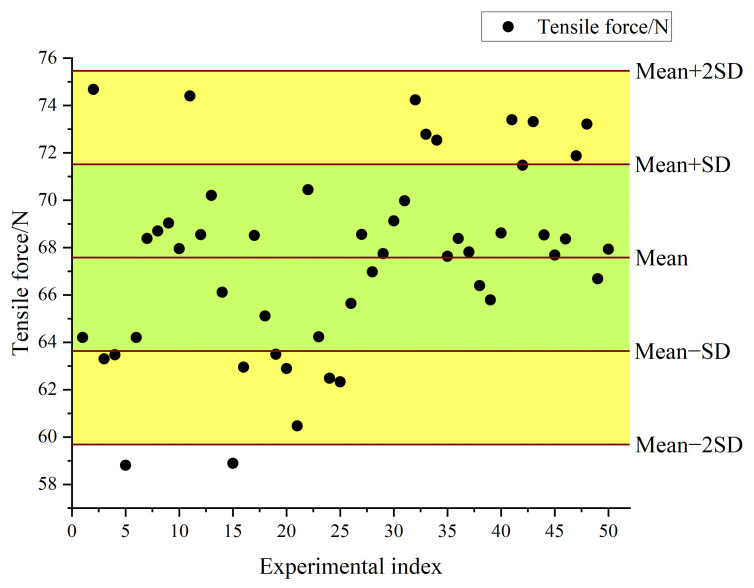
Fifty Repetition Tensile Test Results Scatter Plot of the Silicone Suction Cup (c-3).

Figure 17 presents the scatter distribution of the adhesion forces obtained from 50 repeated tensile tests. The experimental mean adhesion force is 67.576 N with a standard deviation of 3.942 N, corresponding to a 95% confidence interval of 66.46–68.70 N, as shown in Table 5. The range of mean ± 2SD is 59.69–75.46 N, indicating acceptable variability and good repeatability of the measurements.

### 4.3. Comparison Between the Tensile Test Results and the Simulation Result

The adhesion force of the biomimetic suction cup (c-3) measured in the tensile test is presented in Table 5. The average value from fifty repeated tests is 67.576 N. The adhesion force obtained from finite element analysis (FEA) is 72.352 N, calculated using the force check tool in the finite element software under vertical loading conditions, similar to the experimental setup. The difference between the simulated and experimental results is 6.6%, which falls within an acceptable error range. Therefore, the simulation results are considered accurate and reliable.

The silicone material used in the simulation has mechanical properties consistent with the actual material, such as hardness, tensile strength, etc. Therefore, the simulation can reliably reflect the performance of the actual suction cup. For the adhesion force of other suction cups in the study, it can also be calculated through finite element simulation under the same boundary and loading conditions.

## 5. Results

In this study, ten types of biomimetic suction cups inspired by the abalone muscular foot were designed. A comparative analysis of their static performance was conducted to identify the design with the best overall performance, designated as c-3. The introduction of the groove structure effectively improves the stress distribution on the bottom surface, resulting in significant increases in pressure and frictional stress in the groove regions and surrounding areas. At the same time, the overall von Mises stress of the suction cup is slightly reduced. The addition of the sealing ring eliminates the dependence on the outer edge for sealing. It creates a more stable sealing environment, enhancing the reliability of the suction interface. This design prevents inward contraction at the edge of the suction cup and delays the connection between the internal cavity and the external environment. Consequently, the sliding distance is significantly reduced, leading to a marked improvement in the overall adsorption performance of the suction cup.

To quantitatively benchmark the proposed design against the geometrically aligned reference models reported by Xu et al. [29] (Prev-1 and Prev-2) and to place it in context with groove–sealing strategies reported in recent biomimetic vacuum sucker designs [39], a unified performance comparison is summarized in Table 6.

As shown in Figure 14 and Figure 15, compared with the baseline suction cup, the biomimetic suction cup (c-3) shows a 5.9% reduction in maximum von Mises stress, a 498% increase in maximum contact stress, a 498% increase in maximum frictional stress, a 38% reduction in maximum sliding distance, and a 21% increase in maximum outer circumferential deformation.

More importantly, the c-3 design significantly outperforms the circular hole array structure proposed by Xu et al. [29] (Prev-1 and Prev-2) in all interface-related metrics. The contact pressure and frictional stress are increased by more than three times, while the sliding distance is reduced by approximately 35–38%. This indicates that the hexagonal groove-sealing ring composite structure is more effective than the perforated micropore array in facilitating interface load transfer and inhibiting interface slip. Although the maximum total deformation of c-3 is higher than that of the reference model, this does not indicate a weaker structure but rather reflects its greater elastic compliance and surface conformability. The increased deformation capability allows the groove-sealing network to more fully conform to the substrate, thereby enhancing the sealing interface and improving adsorption stability.

## 6. Discussion

The abalone-inspired groove-seal architecture enhances suction performance by optimizing interfacial load transfer. The interconnected groove network creates localized regions of high pressure and friction, while simultaneously reducing the overall von Mises stress. The network redistributes stress more efficiently, rather than simply increasing deformation. Additionally, the peripheral sealing ring forms a stable annular sealing zone that helps maintain vacuum integrity and prevents interfacial slip.

Compared to previous geometric suction designs—such as those based on perforations or holes, and sealing-ring-centered or groove-assisted architectures [29,38,41]—the present study advances passive biomimetic suction by introducing a networked groove pathway with a dedicated sealing boundary. As shown in Table 7, this innovation enhances pressure transfer, shear resistance, and slip suppression simultaneously. Specifically, c-3 shows over three times higher contact pressure and frictional stress, along with a 35–38% reduction in sliding distance. Although c-3 has a higher maximum total circumferential deformation, this primarily reflects greater compliance and conformability, which benefits sealing continuity on non-ideal surfaces and makes it suitable for climbing, perching, and robotic gripping applications.

## 7. Conclusions

This study presents the design of biomimetic suction cups inspired by the structural and functional principles of the abalone muscular foot. The design integrates specialized groove features and a sealing ring and combines finite element analysis (FEA) with experimental validation to optimize structural performance. The main conclusions are summarized as follows:

(1) A comparative analysis of ten biomimetic suction cup structures identifies the design with the best overall performance.
(2) The experimental results verify the reliability of the finite element analysis, demonstrating its effectiveness for structural design and performance prediction of suction cups.
(3) The optimized biomimetic suction cup shows significant increases in pressure and frictional stress, accompanied by a clear reduction in sliding distance, compared to the baseline suction cup (smooth design without grooves or sealing ring). This confirms the superiority of the biomimetic structure in improving adsorption performance, specifically in terms of better distribution of stress and reduced sliding during operation.

It should be noted that the suction cup structure designed in this study is primarily suitable for flat-surface adsorption, and it may face challenges in achieving effective sealing on curved surfaces, presenting certain limitations. Moreover, the suction cup’s operational adaptability is insufficient, and the sealing ring may fail under extreme conditions such as high temperature or mining environments. Future research will focus on optimizing the groove geometry and employing high-temperature-resistant materials to expand its application range and broaden the potential use cases for UAVs and robots. Furthermore, while the nonlinear behavior of the silicone material has been effectively described in this study, the Yeoh model may show some deviation under extreme temperature and deformation conditions. Future work will explore the changes in material behavior under these conditions. Finally, in terms of experimental validation, this study has only conducted preliminary verification of the suction cup’s adsorption performance through finite element simulations and pull-off tests. Systematic sealing performance experiments have not yet been performed. Future research will prioritize evaluating the suction cup’s vacuum retention ability, leakage rate, and sealing durability under long-term and cyclic loading conditions to further validate its sealing performance in practical applications.

## Figures and Tables

**Table 1:** Structural Features and Main Parameters of the Baseline Suction Cup, Literature Reference Designs, and the Proposed Biomimetic Suction Cups. Note: The suction cup designs denoted as a, Prev-1, and Prev-2 are taken from Xu et al. [29], while all remaining designs are developed in this work.

Type	Surface Feature Description	Main Parameters
a	Smooth baseline suction cup	Without grooves or sealing rings (ϕ 55 mm; baseline geometry for benchmarking)
Prev-1	Circular hole array × 50	ϕ 0.5 mm, depth 0.25 mm
Prev-2	Circular hole array × 40	ϕ 1.5 mm, depth 0.75 mm
a-1	Slotted groove array × 40	Width 1 mm, depth 0.5 mm
a-2	Radial groove array × 40	Width 1 mm, depth 0.5 mm
a-3	Herringbone groove array × 20	Width 1 mm, depth 0.5 mm
a-4	Hexagonal groove array × 20	Width 1 mm, depth 0.5 mm
b-1	Sealing ring × 1	Width 0.5 mm, height 0.5 mm
b-2	Sealing ring × 1	Width 1.5 mm, height 0.5 mm
b-3	Sealing ring × 1	Width 2.5 mm, height 0.5 mm
c-1	Hexagonal groove array × 20 + sealing ring × 1	Combination of a-4 and b-1
c-2	Hexagonal groove array × 20 + sealing ring × 1	Combination of a-4 and b-2
c-3	Hexagonal groove array × 20 + sealing ring × 1	Combination of a-4 and b-3

**Table 2:** Structural Characteristics of the Adsorption Plates.

Model	Diameter	Thickness
1	ϕ 65 mm	1 mm
2	ϕ 65 mm	2 mm
3	ϕ 65 mm	3 mm
4	ϕ 65 mm	4 mm
5	ϕ 65 mm	5 mm

**Table 3:** Peak Response Metrics of the Baseline and Biomimetic Suction Cups.

	Maximum von Mises Stress (MPa)	Maximum Pressure (MPa)	Maximum Frictional Stress (MPa)	Maximum Sliding Distance (mm)	Maximum Total Circumferential Deformation (mm)
a	6.768 × 10^−2^	0.762 × 10^−2^	1.524 × 10^−3^	1.401	1.409
a-1	6.455 × 10^−2^	1.659 × 10^−2^	3.318 × 10^−3^	1.389	1.398
a-2	6.691 × 10^−2^	1.953 × 10^−2^	3.906 × 10^−3^	1.417	3.044 × 10^−4^
a-3	6.601 × 10^−2^	1.716 × 10^−2^	3.431 × 10^−3^	1.383	1.391
a-4	6.430 × 10^−2^	2.134 × 10^−2^	4.268 × 10^−3^	1.382	1.392
b-1	6.257 × 10^−2^	3.095 × 10^−2^	6.190 × 10^−3^	1.130	3.748 × 10^−4^
b-2	6.524 × 10^−2^	3.968 × 10^−2^	7.935 × 10^−2^	0.840	4.463 × 10^−4^
b-3	6.433 × 10^−2^	4.528 × 10^−2^	9.055 × 10^−3^	0.796	4.567 × 10^−4^
c-1	6.528 × 10^−2^	2.045 × 10^−2^	4.090 × 10^−2^	1.193	1.398
c-2	6.689 × 10^−2^	4.072 × 10^−2^	8.145 × 10^−3^	0.909	1.688
c-3	6.369 × 10^−2^	4.557 × 10^−2^	9.114 × 10^−3^	0.869	1.702

**Table 4:** Effects of Adsorption Plate Thickness on Suction Cup Performance.

Adsorption Plate Thickness (mm)	Maximum von Mises Stress (MPa)	Maximum Pressure (MPa)	Maximum Frictional Stress (MPa)	Maximum Sliding Distance (mm)	Maximum Total Circumferential Deformation (mm)
1	6.342 × 10^−2^	4.518 × 10^−2^	9.036 × 10^−3^	0.852	1.705
2	6.344 × 10^−2^	4.527 × 10^−2^	9.053 × 10^−3^	0.816	1.705
3 (Original thickness)	6.369 × 10^−2^	4.557 × 10^−2^	9.114 × 10^−3^	0.869	1.702
4	6.340 × 10^−2^	4.525 × 10^−2^	9.051 × 10^−3^	0.752	1.701
5	6.340 × 10^−2^	4.564 × 10^−2^	9.128 × 10^−3^	0.821	1.703

**Table 5:** Comparison between the Tensile Test Results and the Simulation Result of the Biomimetic Suction Cup (c-3).

Parameter	Experimental Test	FEA
Average Force (N)	67.576	72.352
Standard Deviation (SD)	3.942	-
95% Confidence Interval (N)	66.46~68.70	-
Mean ± 2SD (N)	59.69~75.46	-
Relative Error	6.6%	

**Table 6:** Unified performance comparison of the present biomimetic suction cup (c-3) with reference designs.

	Maximum von Mises Stress (MPa)	Maximum Pressure (MPa)	Maximum Frictional Stress (MPa)	Maximum Sliding Distance (mm)	Maximum Total Circumferential Deformation (mm)
a (Baseline)	6.768 × 10^−2^	0.762 × 10^−2^	1.524 × 10^−3^	1.401	1.409
Prev-1	6.656 × 10^−2^	1.057 × 10^−2^	2.113 × 10^−3^	1.370	1.379
Prev-2	6.476 × 10^−2^	1.483 × 10^−2^	2.966 × 10^−3^	1.390	1.396
c-3	6.369 × 10^−2^	4.557 × 10^−2^	9.114 × 10^−3^	0.869	1.702

**Table 7:** Novelty matrix comparing the proposed suction cup with representative groove- and seal-based biomimetic designs.

Study	Groove Topology	Sealing Strategy	Dominant Interfacial Mechanism	Performance Characteristics (Reported/Observed)
[29] (Prev-1/2)	Discrete circular holes/perforations	Rim-based sealing	Primarily local compliance/contact-area modulation	Moderate enhancement in interfacial pressure/friction compared with the smooth baseline
[41]	Bar-type grooves (1D)	Annular sealing ring	Directional load guidance along grooves + sealing-ring-supported vacuum retention	Improved shear resistance; response may show directionality due to 1D groove geometry
[38]	Sealing-ring–centered design (groove networking not the primary variable)	Single-/double-ring sealing; ring number/width/spacing as key parameters	Improved vacuum retention and sealing stability via optimized ring geometry; stress is concentrated around the sealing rings	At 60% vacuum, a double-ring design (1.5 mm width, 3 mm spacing) achieved the highest adsorption force (~15.8% higher than the baseline); higher von Mises stress near the rings and lower stress in the center
This work (c-3)	Hexagonal interconnected grooves (2D network)	Coupled internal annular sealing ring	Network-mediated load sharing (multi-directional) + stabilized annular sealing	Simultaneous improvements in contact pressure, frictional stress, and reduced sliding distance (Table 6)

## Data Availability

The data supporting the findings of this study are available in the Supplementary Materials.

## Supplementary Materials

The following supporting information can be downloaded at: https://www.mdpi.com/article/10.3390/biomimetics11020118/s1, Model and Configuration S1: Finite element analysis (FEA) model, boundary conditions, and ANSYS configuration files used for numerical simulations.
